# An Infrared Laser Sensor for Monitoring Gas-Phase CO_2_ in the Headspace of Champagne Glasses under Wine Swirling Conditions

**DOI:** 10.3390/s22155764

**Published:** 2022-08-02

**Authors:** Florian Lecasse, Raphaël Vallon, Frédéric Polak, Clara Cilindre, Bertrand Parvitte, Gérard Liger-Belair, Virginie Zéninari

**Affiliations:** Université de Reims Champagne-Ardenne, CNRS, GSMA, 51097 Reims, France; florian.lecasse@univ-reims.fr (F.L.); frederic.polak@univ-reims.fr (F.P.); clara.cilindre@univ-reims.fr (C.C.); bertrand.parvitte@univ-reims.fr (B.P.); gerard.liger-belair@univ-reims.fr (G.L.-B.); virginie.zeninari@univ-reims.fr (V.Z.)

**Keywords:** champagne, wine swirling, effervescence, CO_2_ sensor, diode laser spectrometry

## Abstract

In wine tasting, tasters commonly swirl their glasses before inhaling the headspace above the wine. However, the consequences of wine swirling on the chemical gaseous headspace inhaled by tasters are barely known. In champagne or sparkling wine tasting, starting from the pouring step, gas-phase carbon dioxide (CO${}_{2}$) is the main gaseous species that progressively invades the glass headspace. We report the development of a homemade orbital shaker to replicate wine swirling and the upgrade of a diode laser sensor (DLS) dedicated to monitoring gas-phase CO${}_{2}$ in the headspace of champagne glasses under swirling conditions. We conduct a first overview of gas-phase CO${}_{2}$ monitoring in the headspace of a champagne glass, starting from the pouring step and continuing for the next 5 min, with several 5 s swirling steps to replicate the natural orbital movement of champagne tasters. The first results show a sudden drop in the CO${}_{2}$ concentration in the glass headspace, probably triggered by the liquid wave traveling along the glass wall following the action of swirling the glass.

## 1. Introduction

Sparkling French wines from the Champagne region are internationally renowned for their complex aromas and the fineness of their bubbles. Worldwide, champagne tasting is synonymous with festivity and celebration. However, champagne tasting parameters are more subtle and difficult to evaluate for consumers than still wine tasting parameters, mainly due to the presence of dissolved carbon dioxide (CO${}_{2}$) in champagne wines (and more generally in sparkling wines elaborated through the same method, known as Méthode Traditionnelle) [1,2]. According to the Méthode Traditionnelle, a second in-bottle alcoholic fermentation (also known as the Prise de Mousse) is conducted in bottles sealed with a crown cap or a cork stopper [2]. Therefore, as ruled by the so-called Henry’s law, an equilibrium progressively establishes between gas-phase CO${}_{2}$ trapped in the bottle headspace and dissolved CO${}_{2}$ in the liquid phase (i.e., in the wine). At the end of this stage, the partial pressure of gas-phase CO${}_{2}$ reaches close to 6 bars in the sealed bottle (at 12 °C), and the wine is subsequently saturated with a concentration of dissolved CO${}_{2}$ close to 12 g·L${}^{-1}$, which makes champagne one of the sparkling beverages with the highest level of dissolved CO${}_{2}$ [2].

**Figure 1:**
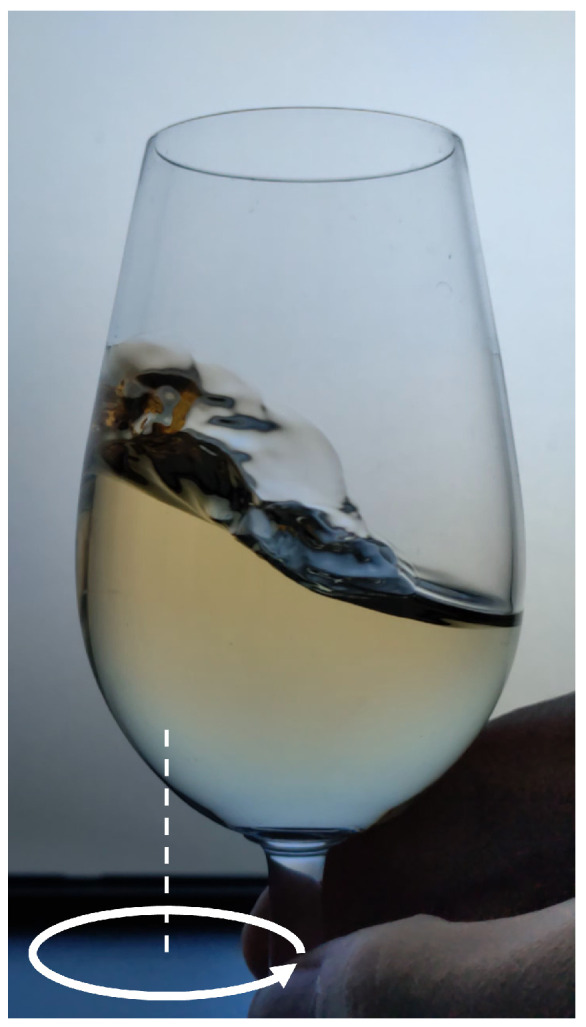
Liquid wave traveling along the sides of a wine glass subsequent to typical manual rotation of the glass conducted with the foot of the glass in full contact with a flat support (desktop).

After uncorking the bottle, the partial pressure of gas-phase CO${}_{2}$ falls, and the liquid phase becomes supersaturated with dissolved CO${}_{2}$ [2]. Therefore, starting from the pouring step, champagne tends to outgas the excess dissolved CO${}_{2}$ by molecular diffusion to reach another thermodynamic equilibrium ruled by Henry’s law [2]. This outgassing process is also responsible for the nucleation and rise of CO${}_{2}$ bubbles (the so-called effervescence) and the subsequent creation of the specific collar of bubbles found at the champagne surface very much sought after by champagne tasters. Moreover, in champagne and sparkling wine tasting, the myriad of bursting CO${}_{2}$ bubbles convey gas phase CO${}_{2}$ in the glass headspace, continuously modifying the chemical space perceived by the consumer through stimulation of the trigeminal receptors [3,4,5]. Above a concentration of 30%, gas-phase CO${}_{2}$ triggers very unpleasant nasal irritation known as carbonic bite [6,7]. Therefore, to better understand the strong interplay between the various parameters at play and to ultimately enhance the champagne tasting experience, monitoring gas-phase CO${}_{2}$ in the headspace of champagne glasses has progressively become a topic of interest over the last dozen years [8,9,10]. Monitoring of gas-phase CO${}_{2}$ in the headspace of champagne glasses under static conditions has highlighted the influence of various parameters, such as the champagne temperature, the glass shape, and the level of champagne poured in the glass [8,11,12]. However, it is noteworthy to mention that while tasting still or sparkling wines, tasters commonly swirl the wine in their glass before inhaling it (Figure 1), with the aim of increasing the release of aromas [13,14,15]. However, the consequences of wine swirling on the chemical space found in the headspace of wine glasses are still unknown.

Among the wide variety of methods used to measure the concentration of gaseous species, spectroscopic methods, and especially methods based on laser absorption spectroscopy in the near infrared region, are effective for sensing gas-phase CO${}_{2}$ [16]. Due to the problem of global warming, monitoring of atmospheric CO${}_{2}$ has become a worldwide interest. In this context, tunable diode laser absorption spectroscopy (TDLAS) was highly solicited, largely due to its high acquisition rate, spectral resolution, reproducibility, calibration-free measurements, and non-invasiveness [17,18,19]. These properties of TDLAS are also needed for monitoring the gaseous headspace of champagne glasses, especially under the swirling conditions triggered by several revolutions of the glass per second around a vertical axis (Figure 1).

In this article, a spectrometer initially developed to measure the concentration of gas-phase CO${}_{2}$ in the headspace of champagne glasses, the so-called CO${}_{2}$-DLS [10], was upgraded to enable a wide range of gas-phase CO${}_{2}$ concentrations in a new homemade orbital shaking setup especially designed to replicate the wine-swirling motion conducted with champagne glasses under standard tasting conditions. The first datasets recorded thanks to this setup are presented.

## 2. Instrument Design

The experimental setup used to monitor gas-phase CO${}_{2}$ in the headspace of champagne glasses is an upgrade of the CO${}_{2}$-DLS previously developed at GSMA [10,11,12]. Based on a convoluted TDLAS design [19,20,21], the two antimonide laser diodes (operating, respectively, at 2.004 µm and 2.682 µm) managed to go through the glass quasi-simultaneously. By scanning distinct absorption strength lines from CO${}_{2}$ rovibrational bands, the two laser diodes were able to cover the whole range of gas-phase CO${}_{2}$ concentrations likely to be found in the glass headspace (ranging from about 0.5 to 100%); for more details, see Table 1. In the original CO${}_{2}$-DLS design, the switch between the two laser was mostly manually triggered by a pneumatic beam shutter [10]. This switch was applied to match the laser concentration range with the CO${}_{2}$ concentration in the glass headspace. Beam shutter activation was performed by visually estimating the absorption depth and was therefore not suitable for fast variations of CO${}_{2}$ concentration. Currently, the new switch system (realized with a galvanometric mirror, see underneath) allows both lasers to monitor the gas-phase CO${}_{2}$ in the glass headspace quasi-simultaneously. Thus, the combination of the two laser measurements makes it possible to observe significant and rapid variations in the gas-phase CO${}_{2}$ concentration throughout the glass headspace monitoring without loss of data.

**Figure 2:**
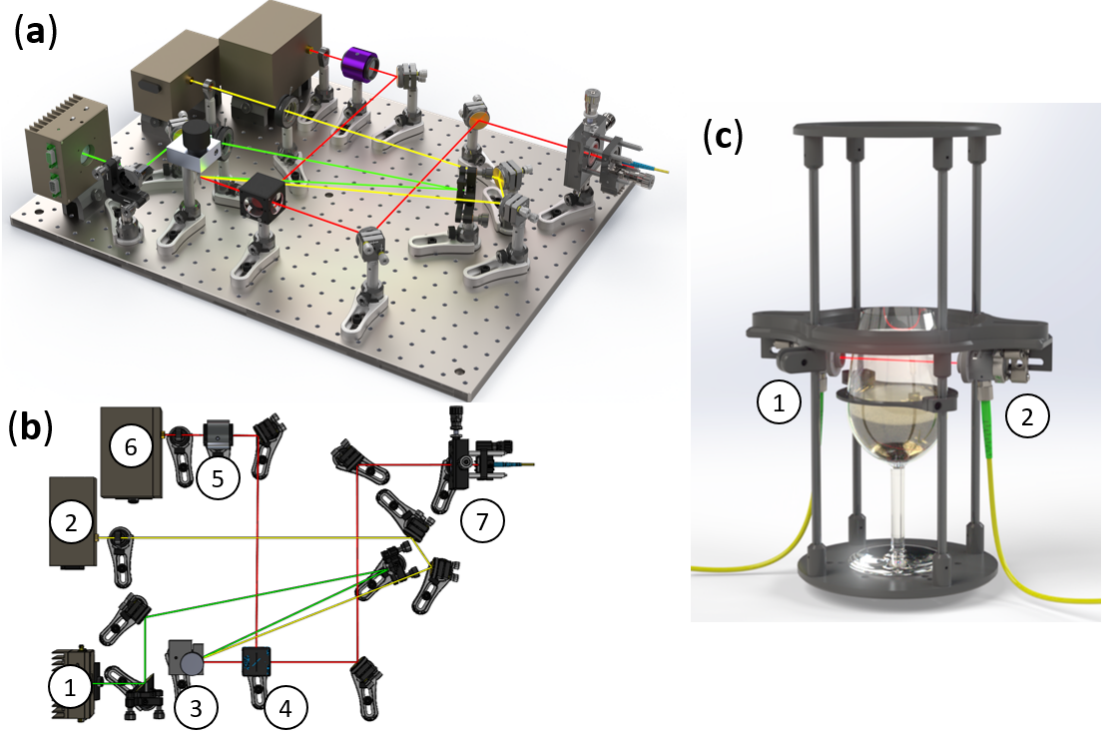
A 3D overview of the experimental spectrometer used for shaping and guiding the lasers’ beams (**a**). Top view of the spectrometer with the corresponding legends: ➀ Laser @ 2.004 µm; ➁ Laser @ 2.682 µm; ➂ Galvanometric mirror; ➃ Pellicle beam splitter; ➄ Germanium Fabry–Pérot; ➅ InAs photodiode; and ➆ Fiber coupler (**b**). The 3D-printed structure used to hold the laser beam position through the champagne glass under swirling conditions with the corresponding legends: ➀ Input reflective collimator; and ➁ Output reflective collimator (**c**). The connection between the two setups is achieved through an optical fiber.

**Figure 3:**
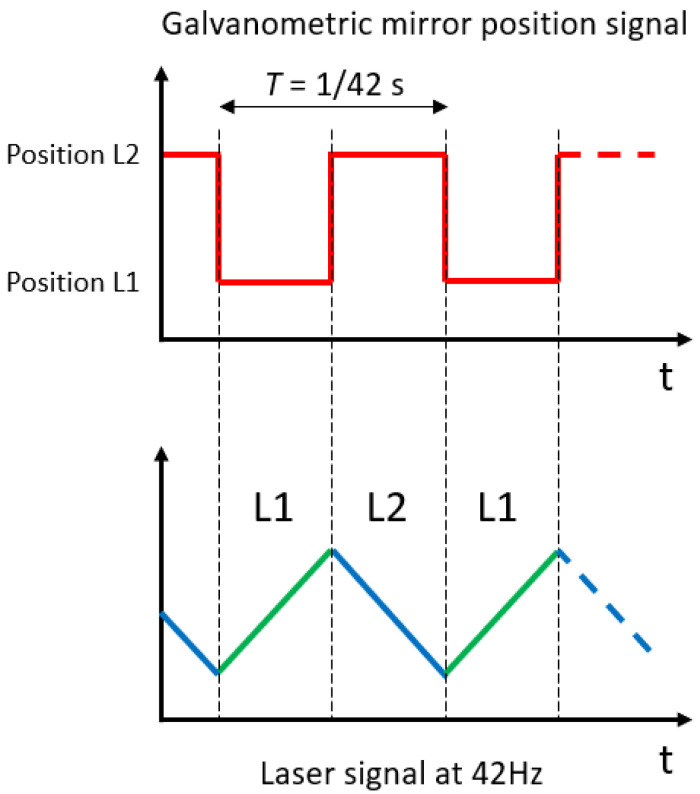
Schematic sketch of the synchronized galvanometric mirror position with the laser wavelength modulation signals.

The whole experimental setup is composed of two parts. The first part is dedicated to controlling the laser sources and managing the laser beams with optical elements (Figure 2a,b). As it is an upgrade of a previously developed CO${}_{2}$-DLS (first designed in 2018), the two-laser collimation and the control device remained unchanged [10]. The main upgrade and development were achieved through the use of a galvanometric mirror (Thorlabs GVS001) upon which the two laser beams converge (marked with label 3 in Figure 2b). This motor-mounted mirror quasi-instantly provides a single path for the two lasers by adjusting the mirror angle and the subsequent laser beam reflection. The galvanometric mirror position switch happens at the half period of the lasers’ current sawtooth modulation (Figure 3). Thus, the final sawtooth modulation is now a combination of the rise from one laser and the fall from the other laser. In order to accurately synchronize each laser’s current modulation with the galvanometric mirror angle switch, these three signals are managed by the same internal clock (a National Instrument NI-7852R FPGA (field-programmable gate array) card). The angular deviation of the galvanometric mirror was adjusted in order to have a short deviation to reduce the transition time between the two positions while maintaining large enough deviation to avoid beam superposition. To satisfy these two conditions, the optimal angular deviation for the galvanometric mirror to change the outcoming laser beam is 1.69°. The lasers’ common path is split by a 45/55 pellicle beam splitter to provide two distinct paths: the reference path operated with a one-inch uncoated germanium Fabry–Pérot (FP) etalon (Laser Component L5940) (marked with label 5 in Figure 2b) and a gas-sensing path where the lasers’ beams are sent through an APC (angled physical contact)/FC (ferrule connector) flurorozirconium single mode optical fiber thanks to a fiber coupler (marked with label 7 in Figure 2b). To avoid absorption by ambient CO${}_{2}$, the entire setup part is enclosed in a PMMA (poly(methyl methacrylate)) box filled with gas-phase nitrogen [10].

The second part of the experimental setup is a homemade optomechanical orbital shaker especially designed to replicate the wine taster’s gesture, which consists of swirling the glass to better express the wine’s bouquet. A direct current motor (RS PRO 834-7663) controlled with a MOTORplate-R1.1 Pi-Plate mounted on a Raspberry Pi 3B+ was used to provide a perfectly circular motion. Moreover, thanks to flat springs and a roller bearing, the plate connected to the motor can rotate without spinning around its axis of revolution, which is a crucial condition for correctly swirling the glass. In addition, a structure designed to support the optical elements is fixed on the plate and surrounds the glass like a rigid exoskeleton (Figure 2c). This element of the experimental setup is made of PLA (polylactic acid) and was produced through 3D-printing to minimize the weight of the structure. Two reflective collimators are mounted on this structure. The first one (Thorlabs RC08PC-P01) is used to project the outcoming laser beam through the glass headspace. The second one (Thorlabs RC08APC-P01) allows the laser light to be injected and guided into another APC/FC flurorozirconium single-mode optical fiber, where the output (assured by a reflective collimator (Thorlabs RC08PC-P01)) is directed to a liquid-nitrogen-cooled InAs photodiode (Judson J12D-M204-R01M). Because this part of the experimental setup is in motion, the flexibility of the optical fibers allowed them to maintain the location of relevant elements with respect to the the glass headspace and to restrict laser absorption by ambient CO${}_{2}$.

## 3. Instrument Control and Data Processing

Laboratory software was programmed in LabVIEW to interface with the driving National Instrument NI-7852R FPGA card used to control the two lasers in both temperature and current sawtooth modulation and to control the galvanometric mirror position. The same LabVIEW software was also used to acquire the data in a customized numerical format file thanks to a National Instrument NI PXI-5105 card. The data were stored in this format to allow high speed streaming while limiting the disk footprint. Three signals were recorded simultaneously: both laser signals from the Fabry–Pérot path and from the experimental path (through the glass headspace), as well as the signal reporting the position of the galvanometric mirror. Because the position of the galvanometric mirror is synchronized with the lasers’ modulation (Figure 3), these data were used to split the ramps from each laser into dedicated files.

**Figure 4:**
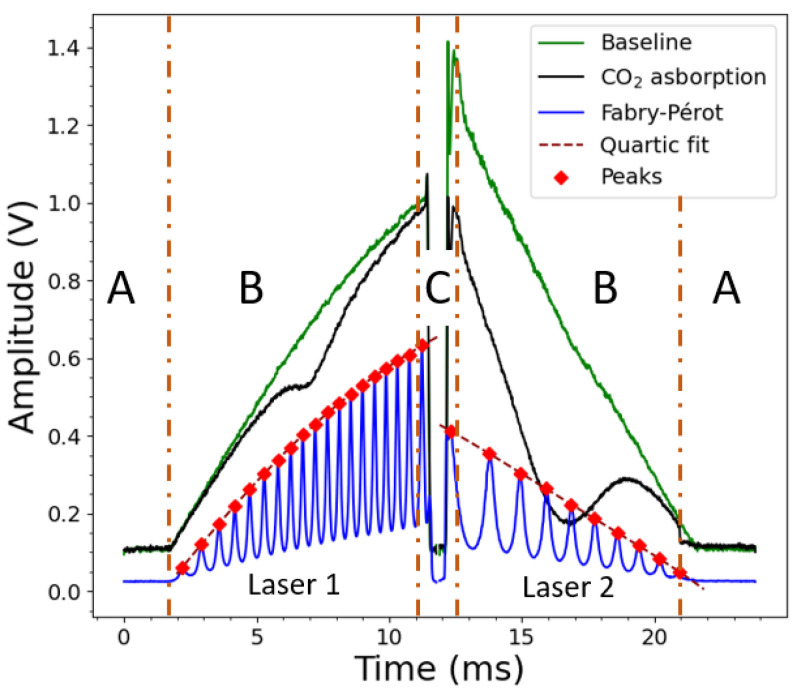
Example of data recorded by Laser 1 @2.004 µm and Laser 2 @2.682 µm in one modulation time period. The black and green solid lines are the baseline and the CO${}_{2}$ absorption raw signals, respectively, recorded by the photodiode along the experimental path. The blue solid line is the Airy’s function signal obtained through the (FP) etalon. The red diamonds are the peaks found with the FP signal to achieve a wavenumber scale thanks to a fourth-degree polynomial function (quartic fit), shown as a red dotted line.

Figure 4 shows a typical time-series data recording, which can be divided into three parts (namely A, B, and C). Part A corresponds to the current below the laser threshold. Because there is no laser light, this part is used to remove the photodiode dark current from the treated signal. Part B is the signal zone used to define the light intensity through the glass headspace. As long as the glass is empty, the resulting light intensity is the baseline ($I_{0}$). As soon as champagne is poured into the glass, the CO${}_{2}$ released in the glass headspace partially absorbs the light from the lasers. Therefore, the subsequent light emerging from the glass indicates the intensity of the transmitted light (*I*). Finally, Part C (which is not used in data processing) is the data acquired between the transitions of the galvanometric mirror.

Data processing is divided into two stages: one stage for each signal. The Fabry–Pérot (FP) signal is used to transform a timescale into a relative wavenumber scale. Light transmission through the FP etalon leads to an Airy distributed signal, where the peaks are separated from each other by the same free spectral range (FSR) given by Equation (1). Therefore, peak detection in the FP signal allows us to obtain a relationship between the time domain and the relative wavenumber domain thanks to FSR incrementation along each peak. Afterwards, a 4th-order polynomial fit is performed in order to obtain a relative wavenumber scale over the entire modulation period.

(1) $$FSR=\frac{1}{2L\left(n\left(\lambda ,T\right)-\lambda \frac{\partial n}{\partial \lambda }\right)}$$

With:

- *L*: cavity length (2.54±0.05 cm);
- *n*: crystal optical index (for germanium n≈4.1 at 2.0–3.0 µm range and 20 °C [22,23]);
- λ: operative wavelength in µm.

Temperature has low influence on the final value. Therefore, the FSRs are roughly 0.045 cm${}^{-1}$ and 0.047 cm${}^{-1}$ for Laser 1 and Laser 2, respectively. The resulting polynomial function is then applied to provide a relative wavenumber scale for the baseline ($I_{0}$) and the transmitted spectrum (*I*). Therefore, as the measurements were conducted at atmospheric pressure, the transmission spectrum ($I/I_{0}$) follows the well-known Beer–Lambert law with a Lorentzian line shape, as defined hereafter:

(2) $$\frac{I}{I_{0}}\left(\sigma ,T,P\right)=\exp\left(-LN\left(T,P\right)\rho \left(\sum_{i}s_{i}f_{i}\left(\sigma ,P\right)\right)\right)$$

(3) $$N\left(T,P\right)=\frac{P}{k_{B}T}$$

(4) $$f_{i}\left(\sigma ,P\right)=\frac{1}{\pi }\frac{\gamma_{l}\left(P\right)}{{\left(\sigma -\sigma_{0,i}\right)}^{2}+\gamma_{l}{\left(P\right)}^{2}}$$

(5) $$\gamma_{l}\left(P\right)=\frac{P}{P_{0}}\left(\gamma_{air}\left(1-\rho \right)+\gamma_{CO_{2}}\rho \right)$$

With:
• σ: wavenumber (cm${}^{-1}$)• $\gamma_{air}$: air broadening coefficient (cm${}^{-1}$·atm${}^{-1}$)• *L*: optical path (cm)• $\gamma_{CO_{2}}$: CO${}_{2}$ broadening coefficient (cm${}^{-1}$·atm${}^{-1}$)• $k_{B}$: Boltzmann constant (J·K${}^{-1}$)• $\sigma_{0,i}$: line center (cm${}^{-1}$)• *T*: temperature (K)• $s_{i}$: line strength (cm${}^{-1}$·(molecule·cm${}^{-2}$)^−1^)• *P*: pressure (Pa)• ρ: CO${}_{2}$ mixing ratio• $P_{0}$: atmospheric pressure (Pa)


Despite the lasers recording one absorption line in the experimental transmission spectrum (Table 1), Beer–Lambert numerical fitting takes multiple absorption lines into consideration. These other lines, spectrally located near the recorded line, influence the line profile (Table 2 shows the line wavenumbers and strengths for both lasers). A Python program was used to adjust fitting parameters, namely the CO${}_{2}$ mixing ratio ρ (ranging from 0 to 1) and the maximum of absorption on a relative scale (to obtain an absolute wavenumber scale). During the validation phase, for the first CO${}_{2}$-DLS development [10], the measured mixing ratio precision was estimated by analyzing the known standard CO${}_{2}$ concentration. For gaseous mixtures with a concentration of CO${}_{2}$ ranging from 2.5 to 100%, the precision was found to vary between 0.001% and 0.4% [10].

## 4. Wine-Swirling Application

### 4.1. Champagne Samples

A standard commercial brut-labeled Champagne wine (with 12.5% alcohol by volume) provided by Champagne Castelnau (Reims, Marne) was used for this set of experiments. One day before the experiments, the bottles were stored in a thermoregulated wine cave, at 12 °C. After each pouring step, the bottles were resealed with a standard champagne bottle stopper and restored in the wine cave until the next pouring step. A concentration of dissolved CO${}_{2}$ of 10.9 ± 0.2 g·L${}^{-1}$ was measured in this brut-labeled Champagne wine by using potentiometric titration conducted on three bottles from the same batch [24].

### 4.2. Glass Shape and Standardized Effervescence

The well-known INAO glass, used worldwide by most professional wine tasters and certified by the Institut National des Appellations d’Origines, was used for this set of experiments. The elongated egg shape of its bowl is indeed well-adapted for still wine or sparkling wine tasting.

**Figure 5:**
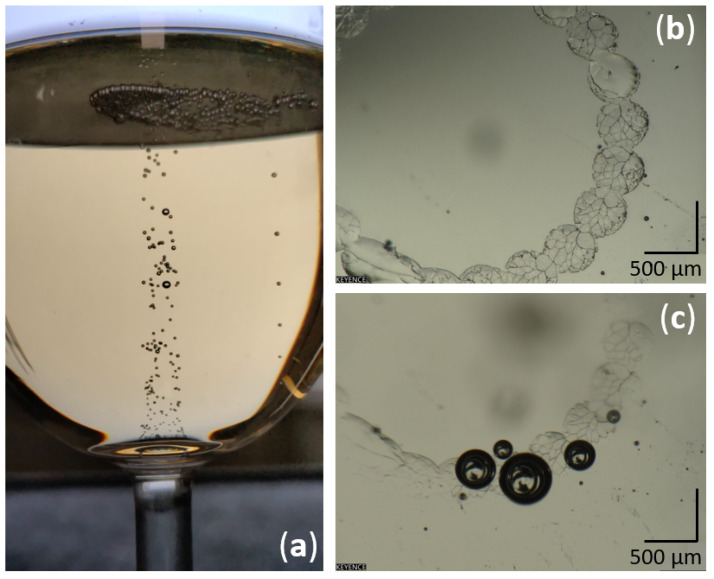
Close-up photograph of the laser-etched INAO glass containing 100 mL of champagne and releasing bubbles through non-classical heterogenous bubble nucleation (**a**); micrograph of the ring-shaped laser etches as observed through optical microscopy (Keyence) of an empty glass (**b**) and in a glass containing with champagne with bubble nucleation captured (**c**).

To trigger a standardized effervescence identical from one glass to another, as shown in Figure 5, all INAO glasses were laser-etched on the bottom with a characteristic ring-shaped structure using 20 successive laser-beam impact points, as described in previous articles [12,25]. Before each set of experiments, glasses were thoroughly washed with a dilute acetic acid solution (10% vol/vol) in order to get rid of any residual nucleation sites likely to trigger bubble nucleation anywhere other than on the laser-etched ring-shaped structure. Glasses were then rinsed with distilled water and dried in a drying oven at 60 °C.

### 4.3. Wine-Swirling Motion Parameters

A recent study showed that the surface-wave dynamics of a liquid in orbitally shaken cylindrical containers depend on the four following parameters: (1) the diameter of the container, (2) the height of the liquid at rest, (3) the orbital diameter of the container, and (4) angular speed [26]. It is worth noting that the same four parameters can also fully define the outcoming surface wave traveling along the wall of a wine glass in orbital motion (with the foot of the glass in full contact with a flat support, as shown in Figure 1). The first two parameters are directly linked to the shape of the glass and the level of wine poured in the glass, whereas the other two are the dynamic parameters of the glass in motion (which might be different from one taster to another).

In order to obtain realistic results, a preliminary statistical study (data not published) was conducted to determine the magnitudes of both the radius of gyration and the angular speed of an INAO glass under standard swirling conditions. To do so, a panel of more than 80 people was recorded while swirling an INAO glass filled with 100 mL of distilled water. The panel was composed of both amateur tasters and wine experts, such as enologists, students in enology, sommeliers, and cellar masters. It is noteworthy to mention that tasters from the panel hardly followed a perfectly circular orbit when swirling the INAO glass. An elliptical trajectory was thus more realistic to describe the motion of wine swirling, with the mean angular velocity and semi-major and semi-minor axis amplitudes and their respective standard deviations (std) given in Table 3.

To replicate as closely as possible the elliptical manual trajectory of tasters swirling INAO glasses filled with 100 mL of distilled water, we arbitrary decided to choose a radius of gyration for the homemade orbital shaker identical to the mean semi-major axis of the panel (i.e., 4.5 mm). Actually, the experimental radius of gyration was 4.51 ± 0.01 mm because the motor gears were manually machined, and it was easier to produce a piece for a larger radius than a piece for a smaller one. Similarly, the angular speed was set to 225 ± 2 rpm (revolutions per minute), close to the mean angular speed of the panel.

### 4.4. Gas-Phase CO${}_{2}$ Monitoring

The homemade orbital shaker was especially designed and developed to highlight the monitoring of gas-phase CO${}_{2}$ in the headspace of champagne glasses under standard swirling conditions. It is worth noting that the wine interface is highly deformed under orbital shaking conditions due to the surface wave traveling along the glass wall (as seen in Figure 1). For correct gas-phase CO${}_{2}$ monitoring under wine-swirling conditions, the laser beam must evidently be above the highest surface wave amplitude in the glass headspace. As in previously published sets of experiments conducted under static conditions [11], it was thus decided to position the laser beam 5 mm below the glass edge (i.e., several mm above the highest position of the wine’s surface wave) in a zone where the nose of champagne tasters inhales the headspace of the glass to perceive the wine’s bouquet. The optical path corresponding to the diameter of the glass 5 mm below the INAO glass edge is 4.6 cm. All INAO glasses had a line drawn on them to mark the 100 mL level of champagne to be poured. Champagne was carefully poured in the glass to avoid the liquid crossing the laser beam (every pouring lasted roughly 15–20 s). When the volume of 100 mL was poured and at rest in the glass, the laser beam traversed the headspace 4.5 cm above the champagne interface. At such a distance from the champagne interface, the collar of surface bubbles and the tiny droplets of wine projected by bursting bubbles cannot impact the laser light and therefore do not affect monitoring of gas-phase CO${}_{2}$. A Python script was executed on the Raspberry Pi to control the orbital shaker motor at the end of the pouring phase. The aim of this program was to pilot five successive 5 s rotation steps, regularly triggered all along the first 5 min following the end of the pouring process. The first 5 s rotation step was triggered by the Python program 30 s after the end of the pouring process, whereas the four following ones were separated from one another by four 1 min time periods.

**Figure 6:**
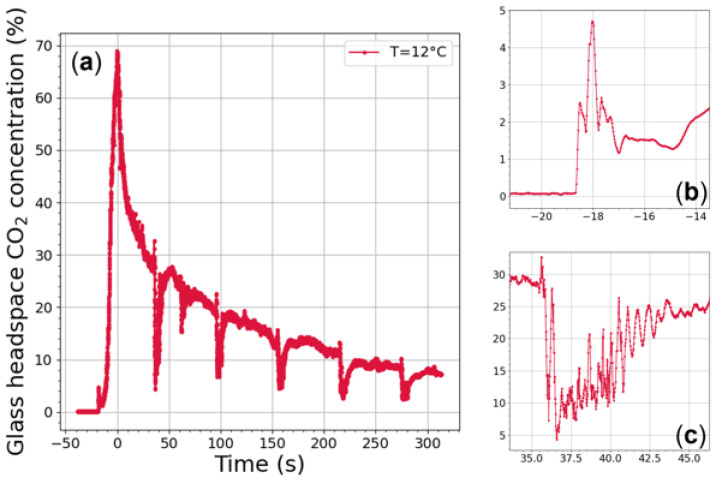
Monitoring of gas-phase CO${}_{2}$ concentrations (in %) conducted 5 mm below the edge of the INAO glass filled with 100 mL of champagne (at 12 °C). During the first 5 min following the end of the pouring step (marked as t = 0 s), five 5 s consecutive rotations (at 225 rpm with a radius of 4.5 mm) were triggered by the orbital shaker, with a 1 min time interval between swirling periods (**a**). Just before champagne falls in the glass, a zoom of the data recording shows a little peak of gas-phase CO${}_{2}$ with a concentration close to 4–5% (**b**). Another zoom highlights the quick variations to CO${}_{2}$ concentration during the 5 s swirling period and the several seconds following (**c**).

**Figure 7:**
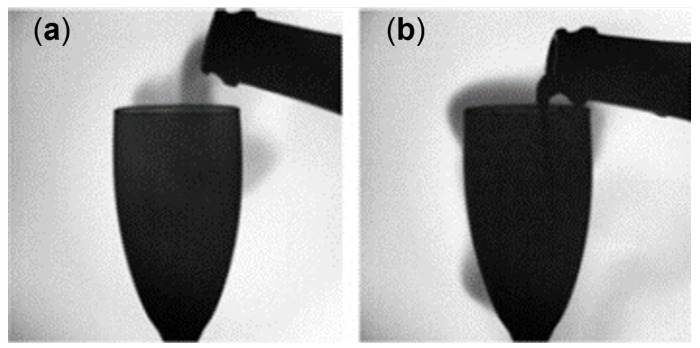
Infrared snapshots, adapted from Liger-Belair et al. [27], showing the behavior of gas-phase CO${}_{2}$ during pouring of champagne in a flute. While tilting the champagne bottle, some gas-phase CO${}_{2}$ (denser than ambient air and initially trapped in the corked bottleneck) falls in the headspace of the flute (**a**) before champagne started to flow through the bottleneck a split-second later (**b**).

Figure 6a shows typical CO${}_{2}$ monitoring as recorded in the headspace of an INAO glass filled with 100 mL of champagne under periodic wine-swirling conditions. Firstly, close observation of Figure 6 reveals a small peak of CO${}_{2}$ rising to almost 5% (between −19 and −17 s), as seen in Figure 6b. This peak was observed for the very first time. It might correspond to gas-phase CO${}_{2}$ (denser than ambient air, and initially under pressure in the headspace of the bottle before uncorking) falling from the bottleneck just before the liquid falls, as observed through infrared imaging in a previous article (see Figure 7) [27]. The snapshots displayed in Figure 7 [27] were taken with a middle-wave infrared camera (CEDIP Titanium HD560M, sensitive to a spectral band between 3 to 5 µm) coupled with a band-pass filter centered on 4.245 µm (the main absorption band of CO${}_{2}$). This technique also involved an extended high-emissivity blackbody used at a controlled uniform temperature of 80 °C and placed approximately 30 cm behind the glass [27].

After this small CO${}_{2}$ peak corresponding to CO${}_{2}$ falling in the glass, a very strong increase in the concentration of gas-phase CO${}_{2}$ is continuously observed during the several seconds of the pouring process, with a maximum CO${}_{2}$ concentration close to 70% at the end of the pouring step. This strong enrichment of gas-phase CO${}_{2}$ in the headspace of the glass is due to the massive loss of dissolved CO${}_{2}$ experienced by champagne during the pouring step, as described in a previous article [28]. Then, an overall decrease in gas-phase CO${}_{2}$ concentration is observed as time proceeds, following global exponential decay-type behavior from 70% to about 30% (thus confirming previous sets of gas-phase CO${}_{2}$ monitoring in the headspace of champagne glasses under static conditions [9,10,11,12]). It is noteworthy that the timescale of this exponential decrease was recently modeled by Moriaux et al. [12] and found to directly depend on the ratio of the headspace volume to the area of the open aperture of the glass.

Thirty seconds after the end of the pouring step, as the glass starts its first 5 s rotation, the CO${}_{2}$ concentration drastically falls below 10% within a second, as shown in Figure 6c. The surface wave triggered by the glass rotation is therefore suspected to force large quantities of gas-phase CO${}_{2}$ out of the glass headspace. Moreover, as highlighted in Figure 6c, CO${}_{2}$ measurements are noisier while the glass is in rotation. Actually, the subsequent, unavoidable mechanical vibrations can slightly change the laser beam injection in the optical fiber, and thus provide an amplitude difference between the transmitted signal from the laser (*I*) and the baseline ($I_{0}$). As the glass rotation stops after 5 s, the CO${}_{2}$ concentration continues to bounce while increasing slowly, leading to damped wave oscillations for about 2 s until the concentration of gas-phase CO${}_{2}$ returns to its exponentially decreasing trend. Such oscillations in gas-phase CO${}_{2}$ concentrations have never been observed in the headspace of champagne glasses under static conditions [11,12]. The same global trend can be observed as the second 5 s rotation starts, and so on until the fifth rotation (but with damped wave oscillations reduced in their amplitude).

## 5. Conclusions

In wine tasting, swirling the wine in the glass before inhaling it is a key step usually performed by tasters to increase the release of aromas in the glass headspace. However, in case of champagne and sparkling wine tasting, gas-phase CO${}_{2}$ progressively invades the glass headspace and triggers a very unpleasant carbonic bite above a concentration threshold close to 30%. The so-called CO${}_{2}$-DLS, developed at GSMA in the past decade to monitor gas-phase CO${}_{2}$ in champagne glasses under static conditions, was adapted and upgraded under dynamic conditions (i.e., for wine-swirling). A galvanometric mirror was added to the spectrometer to perform simultaneous measurements with two lasers (@2.004 µm and @2.682 µm), with the aim of screening the whole range of possible gas-phase CO${}_{2}$ concentrations (i.e., from 0 to 100%) in the headspace of champagne glasses. Moreover, a homemade optomechanical orbital shaker was specifically designed to replicate the natural orbital movement of champagne tasters. A 3D-printed structure surrounding the glass was built to fix the optical elements needed for accurate CO${}_{2}$ measurements in the glass headspace under swirling conditions. The first results unveiled a sudden drop in the CO${}_{2}$ concentration in the glass headspace, probably forced above the glass edge by the champagne surface wave traveling along the glass wall following the action of swirling the glass.

Upgrading the CO${}_{2}$-DLS enabled us to spotlight the very first monitoring of gas-phase CO${}_{2}$ in the headspace of a champagne glass under swirling conditions. Multiple applications of this upgraded CO${}_{2}$-DLS are now expected to provide better understanding of the subtle processes at play during champagne and sparkling wine tasting. Examining, for example, how the gaseous chemical space perceived by sparkling wine tasters is ruled by the swirling parameters, combined with both glass shape and wine temperature, is planned in the near future.

## Figures and Tables

**Table 1:** The rovibrational transition positions in wavenumber with their absorption strength and concentration range under standard tasting conditions for both lasers.

	Laser 1 @ 2.004 µm	Laser 2 @ 2.682 µm
Line scanned (cm${}^{-1}$)	4985.932174	3728.410105
Line strength (cm${}^{-1}$·(molecule·cm${}^{-2}$)^−1^)	1.156 × 10${}^{-21}$	5.792 × 10${}^{-20}$
Concentration range (%)	10–100	0.5–15

**Table 2:** List of the positions in wavenumber (σ) and absorption strength (s) of the lines contributing to the Beer–Lambert fit of the recorded transmissions for both lasers. These data are taken from the Hitran database for a temperature of 296 K.

Laser 1 @ 2.004 µm		Laser 2 @ 2.682 µm	
σ (cm${}^{-1}$)	s (cm${}^{-1}$·(molecule·cm${}^{-2}$)^−1^)	σ (cm${}^{-1}$)	s (cm${}^{-1}$·(molecule·cm${}^{-2}$)^−1^)
4984.526436	1.015 × 10${}^{-21}$	3727.070585	1.071 × 10${}^{-21}$
4985.334523	2.802 × 10${}^{-23}$	3727.082822	5.925 × 10${}^{-20}$
4985.439252	2.598 × 10${}^{-23}$	3727.798746	1.277 × 10${}^{-21}$
4985.932174	1.156 × 10${}^{-21}$	3728.410105	5.792 × 10${}^{-20}$
4986.514153	2.397 × 10${}^{-23}$	3728.557020	1.467 × 10${}^{-21}$
4986.542862	2.201 × 10${}^{-23}$	3729.262969	1.641 × 10${}^{-21}$
4987.308270	1.253 × 10${}^{-21}$	3729.712250	5.514 × 10${}^{-20}$

**Table 3:** Statistical results obtained from a panel of more than 80 people recorded while swirling an INAO glass filled with 100 mL of distilled water. For each parameter, the arithmetical mean is presented together with its standard deviation (std).

Parameter	Mean ± std
Semi-major axis	4.5±2.2 mm
Semi-minor axis	3.2±1.7 mm
Angular speed	226±30 rpm

## Data Availability

Data underlying the results presented in this paper are not publicly available at this time but may be obtained from the authors upon reasonable request.
